# Obstetrics

**Published:** 1857-09

**Authors:** 


### OBSTETRICS.

1. SECONDARY UTERINE HEMORRHAGE. *By Thomas F. Cock.*—The subject of secondary hemorrhage from the uterus has claimed less of the attention of systematic writers upon obstetrics than its importance and frequency demand. The term "secondary" is designed to include such considerable losses of blood as occur between the periods of some six hours after labor is completed and a number of days, varying from eight to thirty.

Such hemorrhages are of importance, because, in many cases, they have proved fatal; and their treatment demands some peculiarities beyond that usually pursued in flooding. Roberton has directed attention to this question in his excellent *Treatise on Diseases of Women*, etc., illustrating it by cases, but for the most extended and satisfactory notice, we are indebted to Dr. A. H. McClintock, who published in the May number, for 1851, of the Dublin *Quarterly Journal of Medical Science*, a valuable paper which contains the best digest of the subject. In relating the accompanying cases, I shall simply follow the etiology which Dr. McClintock has sketched, illustrating the subject by such occurrences as have come under my own notice.

Of the various *causes* of secondary hemorrhage, none is more frequent than that of a *retention of a portion of the placenta*, when manual separation has been practised for adhesion. This form is well described in books, and the danger of the case arises less from the amount of blood lost than from the liability to purulent absorption caused by the presence of a putrid mass in the uterus. The disposition to absorption is also increased by the loss of blood the patient has sustained. The following cases illustrate these points:

*Case* 1.—A multipara; æt. 28 years; entered the N. York Hospital, October 29, 1856, in labor with her fourth conception. Her previous labors had been lingering, and she had had floodings. She was delivered on the 30th, and hemorrhage ensued from atony of the uterus. The placenta was partially removed, portions having remained attached to the uterus. I saw her for the first time, on the fourth day after labor, and found her exhibiting in a marked degree the effects of loss of blood; portions of the placenta had been expelled, with hemorrhage. By examination, I found a portion of placenta within the grasp of the os uteri, and removed it; hemorrhage recurred the next day, and under the reiterated losses the woman succumbed. On *post-mortem* examination, a piece of placenta was found near the fundus of the firmly-contracted uterus; pus was found abundantly in one ovary.

On several other occasions, after manual extraction of the placenta, I have had hemorrhage, but none of serious import. Two autopsies, in addition to the above, have shown pieces of retained placenta, giving rise to phlebitis.

2. A *retained coagulum* acts similarly, and is a source of danger so long as it remains. The nucleus of such a clot may be a portion of membrane, retained by adhesion, which is not unfrequently so firm as to cause considerable inconvenience to detach it.

3. *Relaxation of the tonic contraction* of the uterine tissue may obtain for some days after labor, and if this be combined with any one of the circumstances which hurry the circulation, hemorrhage would be likely to ensue. And these circumstances may properly be reckoned under a fourth head:

The *incautious use of stimulants; premature exertion; agitation of mind,* are frequent, perhaps the commonest, causes of flooding subsequent to labor. The additional impetus to the circulation overcomes the constriction exercised by the uterine fibres on the sinuses, and hemorrhage ensues.

*Case* 2.—A primipara was confined on March 3, 1856, with a natural but rather tedious labor. On the 13th, ten days after, she was dressing her child, and observed something about its navel which alarmed her. She was at once seized with hemorrhage, which prostrated her considerably. Ergot and elixir of vitriol were given, and the bleeding ceased, but there remained marked tenderness in the right iliac region, and a frequent pulse. Before confinement, a pain had existed in this situation. On the 21st, while she was sitting up, the bleeding recommenced, and from its effects she fainted. To control this, the alum plug was used, and ergot given with gallic acid, internally. No further return of bleeding was noticed in this case, but convalescence was tedious and prolonged, and some months elapsed before she regained her strength.

*Case* 3.—A primipara was confined June 5, 1856, and did well until the 23d, being able to wash during this day. In the evening she received some intelligence which disturbed her, and, while talking to some friends, was seized with flooding, which caused faintness. Ergot and gallic acid, aided by rest, controlled the bleeding, and the use of the alum plug (which was prepared), was not required. On the

28th, a slight recurrence was noticed, but required no special attention. On July 3d, she went to the country.

*Case* 4.—A multipara was confined on July 22, 1856, after a labor of moderate severity, and was able to sit up on the 28th. The 31st was a very warm day, and the patient was fatigued by having the sole care of the child. While so engaged, she became faint, and lost considerable blood, by which she was much alarmed Tinct. of ergot with tinct. of matico was directed, and an alum plug prepared for an emergency, but there was no return of hemorrhage.

*Case* 5.—This case has been reported in a previous number of this journal, and is introduced here simply for the purpose of grouping cases exhibiting similar phenomena. A primipara was delivered May 12, 1848. She had a slow getting up, and while engaged in washing, on June 1, had some bloody discharge. On June 4, she went to church to have her child baptized, and, while standing during the administration of the ceremony, flooding came on. She continued to stand until she became faint, and was carried home. After the application of ice and a bandage, the flooding ceased. Two days after, while visiting her, I found her lying prostrate on the floor, pale and faint, and with some hemorrhage. She had risen from her bed to get something, had fainted and fallen on the floor. This patient was exceedingly imprudent in exertion, but finally recovered after a tedious convalescence.

*Case* 6.—Was seen at the Lying-in-Asylum, in connection with Dr. Fergusson, then resident physician, by whom it has been reported. A primipara was seized on the thirteenth day after labor with flooding, resulting, as the Doctor supposed, from premature removal of the binder. She had flooded considerably, and was quite reduced. Cold, ergot, acetate of lead with opium, and the readjustment of the binder, sufficed to restrain the bleeding.

5. *Constipation*, by the obstruction to the circulation produced upon the left iliac vein, may also be a source of bloody discharge from the uterus.

6. *Functional disorder of the liver* is said, also, to play a part in such hemorrhages.

7. Bennett claims no small share for *ulceration of the cervix* as a cause for draining from the uterus after labor, but though recognizing the condition as one likely to produce a continuous flow, in no case have I seen decided hemorrhage resulting from such abrasions.

8. *Polypus of the uterus* unquestionably gives rise to hemorrhage as connected with labor. The only case I have seen of this complication presented flooding as immediately following the expulsion of the placenta. It has been previously published.

*Case* 7.—In May, 1852, I was called to see, in Bellevue Hospital, a primipara, flooding after delivery. The labor had been normal, the placenta being spontaneously expelled; flooding followed, the uterus being well contracted. On inserting the finger between the labia, a tumor was found, which was ascertained to be attached to the uterus, in size and shape, much resembling the virgin uterus; considerable loss of blood occurred—about two quarts. Ergot and firm pressure over the uterus failing to check the discharge, the hand was introduced into the uterus, and then it ceased. Three weeks after, by speculum examination, the tumor was found to have diminished to a mere excrescence on the cervix.

Somewhat resembling this case was one in which an elongated anterior lip of the cervix, seemed to be the producing cause of a hemorrhage. In a subsequent labor of this patient, when this prolongation did not obtain, there was no marked loss of blood.

9. *Inversion of the uterus* is a well-recognized cause of this peculiar variety of flooding; but of this accident, the only instance I have seen was in a case which Dr. Van Pelt has reported in the New York *Medical Times* for February, 1853. No difficulty was experienced in restoring the uterus to its natural position in this case, and no hemorrhage ensued. The knowledge of the possibility of inversion as a cause of secondary hemorrhage will induce a strict scrutiny in all such cases; and manual examination is indispensable, both for diagnosis and for treatment.

10. *Various injuries* to the uterus, or even the vulva, will sometimes give rise to serious discharges of blood. Such injuries may occur from imprudent or prolonged efforts in instrumental or manual delivery, and are also found in abscesses in the vicinity of the uterus, or even, as has been noticed, an aneurismal sac in the walls of the uterus has been the source of the discharge. Montgomery has described a thrombus near the cervix as causing hemorrhage, and others have related fatal cases from the same cause.

A case is recorded by George Todd, in the London *Lancet* for April, 1857, which proved fatal three weeks after delivery. In commenting on the case, Mr. Todd surmises "that the cause of the flooding is to be sought for, neither in the relaxed condition of the uterus, nor in its post-partum sub-involution," but ratheri nclines to believe that "*an altered condition of the blood* and circulation may play an important part in the causation of the hemorrhage." His patient had had severe confluent small-pox, two months before delivery. (The child it may be remarked was born healthy.)

Bloody uterine discharges are not uncommon with typhus fever.

The following case, though imperfect without the autopsy, may be adduced as an example of secondary hemorrhage from injury.

*Case* 8.—Was called November 8, 1855, to see a multipara, of whom the following history was given:—She had been some time in labor, under the care of a student, and when seen by Dr. Van Pelt was in full labor with a breech presentation. In the absence of the latter, another doctor was called in "who attempted to turn," and on his return, the right arm was protruding from the vulva. Repeated and prolonged efforts at version proving unsuccessful, I was called to see her, and found the patient much exhausted by her own and by her attendants' exertions; the right arm was extruded from the vulva, swollen and livid; beyond this arm and high up was the left foot, and still further up could be felt the other hand. Version was effected with difficulty, and the patient was much exhausted.

On the fifth day after delivery, the student who attended her, while sitting by her, was startled by a noise, and found she was bleeding profusely, the blood being florid. From the effects of this hemorrhage she did not rally, but died on the succeeding day. No autopsy was made. It was supposed that the long-continued efforts at version had produced inflammation, and ulceration of some considerable vessel had followed, giving rise to the hemorrhage.

*Treatment.*—As remedial measures, perfect *rest* in the horizontal position, aided by *pressure* over the uterus, and the use of *ergot*, naturally occur as of service in most cases; *cold,* either by *ice* or *enema,* may also be useful, but the remedy requires some caution, when inflammatory affections are to be apprehended. Of styptic remedies, I have placed the most reliance on *gallic acid,* freely given, preferring it to *acetate of lead* or *tannin;* in some cases of hemorrhages, connected with abortion, *matico* has been conjoined with ergot, and, in this form, also, to a more limited extent, and benefit has ensued.

The one remedy in which I have placed most confidence in the treatment of uterine hemorrhage apart from parturition, is the *alum plug.* In the floodings of abortion, this has proved, I may say, an unfailing resource; and in this special form of secondary hemorrhage, a plug is always left to be used if the discharge recurs. The plug is prepared by shaping a piece of alum to the size of the thumb, through which, by means of a red-hot iron, a hole is bored, through which a string is attached, by which the plug may be withdrawn if it causes too much irritation. The direction is usually given to the patient herself to push this piece of alum as far as possible up the vagina, upon the recurrence of hemorrhage. But one objection to the use of this remedy can obtain, viz.: when the vagina is abraded or ulcerated; in such cases, alum would prove too irritating; perhaps, in such cases, an ordinary tampon of cotton, sponge, silk handkerchief, etc., might be useful, but, during their use, a strict watch must be maintained over the uterus, lest internal hemorrhage should occur.

The treatment of retained placenta, or a clot in utero, is, of course, the removal of the exciting cause, when practicable, but violence in removing is more hazardous than hemorrhage from continuance of the presence of the foreign body.

If the bowels are constipated, or functional disorder of the liver exists, the remedy is obvious.

Ulceration or abrasion of the cervix is controllable by the use of *nit. argent,* locally applied.

Inversion, of course, demands *restitution,* if possible; and, by the aid of chloroform, cases, before deemed irremediable, have been relieved.

The relief of polypus demands careful consideration; the exceeding vascularity of the uterus immediately after labor and its proclivity to phlebitis, makes caution in operative measures essential. *Torsion* and the *ligature* have been most frequently employed; to these *excision* is added. McClintock remarks, "notwithstanding the array of evidence in favor of the early performance of the operation, it still can hardly admit of the question that the safest and most prudent course, in cases of secondary hemorrhage from polypus, will be to forego all attempts at extirpating the tumor as long as possible, or until the woman has recovered from the effect of parturition, when the attendant risk will be infinitely less." "Should hemorrhage persist in spite of cold, styptics, the tampon, and, perhaps, ergot, our only alternative is the removal of the tumor."

In detailing the above cases, a special reference has been had to the paper of Dr. McClintock, and if his essay were generally in the hands of the profession, there would be but little necessity for such details; as, however, it seems to have met with but limited circulation, I have ventured to publish my few cases, in the hope of drawing more attention to the subject, and of eliciting the more extended experience of others in regard to it.—*New York Journal of Medicine,* July, 1857.

2. ON THE USE OF THE SPECULUM. By *Robert Lee.*—The author referred to the tabular statement of two hundred and twenty cases of real and imaginary disease of the uterus, published in the 38th volume of the Medico-Chirurgical Transactions, and presented in a similar tabular form the details of eighty additional cases which had since come under his observation. Of the 300 patients, 47 were unmarried; one had barely completed her 18th year, several were under 20, and the majority under 30 years of age, and were suffering from hysteria, leucorrhœa, dysmenorrhœa, or some nervous affection of the uterus, without inflammation, ulceration, or any structural disease or displacement of the organ. In Case 256 the patient had been told that the womb was prolapsed and much ulcerated, and an instrument had been introduced for six weeks, with an aggravation of all the symptoms. The hymen was found so perfect on examination that it was impossible to reach the os uteri without using an unjustifiable degree of violence. On the ground of morality, and on every other ground, he could see no defence for the employment of the speculum in these 47 cases. Of the 300 patients 70 were barren, and the sterility was not removed nor the other symptoms relieved in a single instance. Several of these individuals spoke with horror and shame of the treatment to which they had submitted. A considerable number of the cases were suffering from cancerous disease, in all of which the symptoms seemed to have been aggravated by the treatment. In Case 236 the character of the disease was unmistakable, but after an examination with the speculum a favorable prognosis had been given, and the actual cautery employed for months, and hopes of recovery held out to the last. The author expressed his conviction, that neither in the living nor in the dead body had he ever seen a case of similar ulceration from chronic inflammation of the os or cervix uteri, and to apply the term to states of the os uteri in which the mucous membrane, or, as it is termed by some, the basement membrane is not destroyed by ulceration, was an abuse of language calculated only to deceive and mislead the members of the Medical Profession, from whom the truth has been carefully concealed. The speculum emanates from the syphilitic wards of the Hospitals at Paris, and it would have been better for the women of England had its use been confined to those institutions.—*Med. Times and Gazette*, June 20, 1857.

3. PRACTICAL OBSERVATIONS ON BLISTERING THE CERVIX UTERI, AS A REMEDIAL AGENT IN THE TREATMENT OF CERTAIN DISEASED CONDITIONS OF THAT VISCUS. By *Robert Johns*, of Dublin.—It is a fact well known to the experienced uterine pathologist, that it is not unusual, even after diseases of the womb have been removed, for some one symptom, most frequently that which throughout the duration of the affection had been most distressing, to remain, resisting all means employed for its removal. In some instances it disappears by degrees after a short space of time, whilst in others the result is not so fortunate, in consequence of which the patient and her medical adviser become mutually discontented at the issue, and she seeks advice in all quarters, but to no avail, until at last she abandons all treatment, and gives herself up a prey to despondency and despair, being fully persuaded that she is suffering from cancer or some such incurable disease; and as her general health remains unimpaired, she is regarded in the light of a hypochondriac by her unsympathizing friends, a state of things far more likely to aggravate matters than alleviate them. As the following remarks from the pen of that eminent uterine pathologist, Dr. James H. Bennet, bear much upon this subject, I think it may not be out of place here to quote them:—" Generally speaking, all uterine pains vanish when the disease of the cervix is cured. This is not, however, invariably the case. The pain in the back, more especially, may remain long after all trace of disease has disappeared from the uterus, varying in intensity without any tangible reason. The repeated application of large blisters will often permanently remove the pain; if not at first successful, their application will generally be found to be so in a few months later."

Now it is to the means of removing these harassing but not dangerous symptoms, as also for the cure of minor diseases of the uterus of a very chronic character, of ovarian irritation, after other more active measures have been employed, and for expediting the cicatrization of some ulcers, I would direct attention; but I must premise by saying, that what I am about to recommend is very simple in its application, is perfectly devoid of danger, either immediate or remote, and is never accompanied nor followed by any alarming symptoms. I do not, however, offer it as a panacea for all uterine complaints, but I wish merely to restrict its employment to the class of cases already detailed, in which I trust it will be as successful in the hands of others as it has been in mine.

From amongst other cases of uterine disease treated by me within the last eight months, I have kept notes of about two hundred and fifty; and in many of them I have pursued the plan of treatment now about to be recommended; but as the symptoms and other details in many of them were similar, and their recital would be tedious, I will only give a short sketch of a few, as a type of the remainder.

As it is familiarly known that leeches, when applied directly to the cervix uteri in inflammatory and other affections of that and neighboring organs, act far more beneficially than when employed externally, and at a greater distance from the seat of the disease (as first enjoined by M. Guilbert),—so, as blisters, when used externally, act sometimes salutarily in the cases now under consideration,—may we not reasonably expect that their direct application to the offending viscus will be still more likely to be productive of good? Actuated by this, as well as other reasons, I have made trial of their efficacy, and I have not been disappointed in my expectations.

The plan I adopt for blistering the cervix uteri is as follows: The parts are first brought into view by means of a speculum,—I generally use Fergusson's, but with moderate care any other will answer as well. They are then to be well freed from any mucous or other discharge by a dry, soft sponge; sometimes the mucus is so adherent, particularly when exuding from the os, that it is necessary to damp the sponge for its removal; in all cases, however, the parts must be well dried; after which a concentrated solution of cantharides in sulphuric ether, mixed with the ordinary solution of gutta percha in chloroform, in the proportion of two parts of the former solution to one of the latter, is to be rapidly rubbed on the cervix by means of a camel's-hair pencil two or three times, according to the effect produced, as indicated by the appearance of the part, or by the sensations of the patient.

My first essay was with vesicating collodion, but as it caused great pain, both during its application and for hours afterwards, only ceasing on the appearing of the watery discharge, and as its operation was not sufficiently expeditious or efficacious, I had a strong solution of cantharides in chloroform, in which gutta percha was afterwards dissolved, prepared for me by Mr. Walsh, of Westland Row, which had the advantage of being painless during and after its use; but it was not so speedy in its action, nor so powerful in its effects as the collodion. Therefore, after consulting with several chemists in the city, I eventually applied to Mr. Williams (Dr. Butler's chemist), who, after having made several experiments, kindly presented me with a specimen of the preparation which I now use, and offer to the prosfession as being fully adequate to fulfil the indications required. It is frequently of advantage to keep open the blistered surface, and this is satisfactorily done by the weaker preparation in chloroform, which I denominate vesicating gutta percha, No. 2,—the ordinary one (vesicating gutta percha, No. 1) being rarely required for that purpose. For some days after the operation I direct vaginal injections of cold water to be used, and sometimes I wash over the parts with a weak preparation of nitrate of silver. At first I was in the habit of keeping open the blisters by some strong caustic, but I very soon learned that the weaker solution of cantharides answered the purpose better.

During the appliance of this remedy the patient experiences a pricking, stinging pain, together with a sensation of heat, sometimes amounting to burning—it is very bearable, and ceases almost immediately, indeed, in some cases she will not tell you of it unless you ask her; in others a sweet, pungent taste and smell are experienced, or an ethereal odor is perceived on her breath by another. Very frequently, in fact generally, small vesications appear at the time, and a watery discharge sets in within half an hour afterwards (which has a scalding sensation whilst passing), sometimes even before the speculum has been withdrawn. This discharge, starching the linen, and in other respects similar to that from blisters externally formed, lasts commonly for three days, when to it succeeds one of a slightly purulent nature, but not productive of pain. At this stage we shall find the epithelium thickened and raised, and coming off in patches, like bits of chewed paper; but, prior to it, vesications, like to those on the skin, are very plainly visible with their exudation. On more than one occasion I have seen a watery discharge produced by preparation No. 2, from a surface previously blistered, used to keep it open; and the same phenomenon has occurred on an ulcerated surface.

Blistering the cervix uteri does not cause any unpleasant sensations towards the rectum, bladder, or neighboring organs. I never saw strangury or such like affection thereby induced; on the contrary, I have employed it more than once when vesical irritation was present, which, so far from being increased, was completely removed by two applications.

The average length of time for repeating this treatment is about six days, unless it be desirable to keep up the process; then, in that case, it would be about three days.

From my experience of the treatment now under consideration, I would draw the following deductions:—

1. That minor idiopathic affections of the uterus and ovaria are curable by blistering the cervix uteri.

2. That symptomatic and sympathetic pains at the decline of uterine and ovarian diseases, and after the cure of those affections, are removable thereby.

3. That ulceration of the cervix uteri sometimes quickly cicatrizes under this treatment.

4. That the phenomena attendant and consequent on blistering the cervix uteri are similar to those produced on other parts of the body.

5. That it is an operation completely devoid of danger, and that it does not cause any unpleasant symptom towards the rectum, uterus, or other neighboring organs.

6. That irritation of the bladder is not necessarily a barrier to blistering the cervix uteri, as this unpleasant symptom is sometimes removed by it.

7. That enlargement of the cervix or body of the uterus from engorgement, or hypertrophy, is not removable by blistering the cervix alone, but that it acts well sometimes in such cases as an adjuvant to other treatment.

8. That the best and most speedy way of blistering the cervix uteri is by a strong solution of cantharides, well and quickly rubbed in with a camel's-hair pencil.

9. That the combination of some sedative or anodyne with the blistering fluid is essential to prevent pain.

10. That chloroform, with gutta percha, is preferable to any other medicament for combining with the blistering fluid, as in the first instance it increases its vesicating powers, and afterwards relieves and removes the pain thereby induced.—*Dublin Quarterly Journal Medical Science*, May, 1857.

